# Psoriatic Skin Lesions Induced by the Bacillus Calmette-Guerin (BCG) Vaccination in a Child With Latent Tuberculosis Infection: A Case Report

**DOI:** 10.7759/cureus.83857

**Published:** 2025-05-10

**Authors:** Yuta Norimatsu, Kazuma Ito, Takayuki Shiomi, Katsunori Fujii, Makoto Sugaya

**Affiliations:** 1 Department of Dermatology, International University of Health and Welfare Ichikawa Hospital, Ichikawa, JPN; 2 Department of Dermatology, International University of Health and Welfare Narita Hospital, Narita, JPN; 3 Department of Pathology, International University of Health and Welfare Narita Hospital, Narita, JPN; 4 Department of Pediatrics, International University of Health and Welfare Narita Hospital, Narita, JPN

**Keywords:** bacillus calmette-guerin (bcg) vaccination, childhood immunization, koch’s phenomenon, latent tuberculosis (ltbi), psoriasis, psoriatic skin lesions

## Abstract

Japan has a policy of vaccination with Bacillus Calmette-Guerin (BCG) and diphtheria, tetanus, and pertussis, and inactivated polio vaccine (DTP-IPV); however, there are rare reports of psoriasis-like skin rash caused by these vaccines. The mechanism by which psoriasis-like skin rash develops after BCG vaccination is unknown, but it has been suggested that it may occur when BCG vaccination is given to patients infected with tuberculosis. Here, we report a case of psoriatic skin lesions following BCG vaccination in a five-month-old girl with latent tuberculosis infection (LTBI). The child received the BCG vaccine and a third dose of DTP-IPV at five months of age in accordance with the vaccination schedule recommended in Japan. Soon after the vaccination, erythema with pustulation developed at the BCG injection site. Erythematous plaques were also seen, mainly on the trunk. Her father had suffered from tuberculosis when the patient was born, and her mother had LTBI. Therefore, the skin rash on the vaccination site was considered Koch’s phenomenon. She was brought to the pediatric department in our hospital to check the infection status of tuberculosis. The tuberculin skin test was positive. A T-cell-based enzyme-linked immunospot assay for tuberculosis was also positive, while there were no abnormalities in the lung field, leading to the diagnosis of LTBI. Treatment with isoniazid was started two weeks after the patient's initial visit to our hospital. Erythematous plaques on the trunk persisted, and the patient was referred to our department six weeks after the initial visit to rule out cutaneous tuberculosis. A skin biopsy revealed psoriasiform acanthosis and Munro's microabscess. No mycobacteria were detected by Ziehl-Neelsen staining, and tissue culture was negative. Therefore, she was diagnosed with psoriatic skin lesions after BCG vaccination. Topical application of maxacalcitol ointment and prednisolone valerate acetate ointment relieved the plaques. No recurrence of skin rash was observed during a five-month follow-up.

## Introduction

Psoriasis is one of the most common immune-mediated inflammatory skin diseases. It can cause arthritis and uveitis as well as skin inflammation [1]. It is associated with metabolic syndrome and, in severe cases, may affect prognosis [2]. Many factors are known to contribute to the development and severity of psoriasis, including stress, smoking, drugs, infection, and alcohol [1,3].

In recent years, it has become known that while the COVID-19 vaccine causes a variety of skin conditions, psoriasis is a relatively common skin condition triggered by it, causing both onset and exacerbation [4]. On the other hand, not all vaccines cause psoriasis relatively well.

Tuberculosis is an infectious disease caused by *Mycobacterium tuberculosis *and is an important infectious disease, one of the top 10 causes of death [5]. In psoriasis, patients with latent tuberculosis infection (LTBI) may develop tuberculosis when immunosuppressive drugs, mainly anti-tumor necrosis factor α inhibitors, are used [6]. It is estimated that more than 10% of patients with moderate to severe psoriasis have LTBI [6]. It has been hypothesized that there may be a concept of tuberculosis-related type of psoriasis, but so far, the mechanism has not been elucidated, and the causal relationship is still unclear [7]. It has been reported that central memory T cells for tuberculosis are deficient in psoriasis, but effector-memory T-cells remain [8]. Therefore, patients with psoriasis may have a false-negative tuberculin skin test, but they are thought to retain immunity to tuberculosis.

Japan has a policy of vaccination with Bacillus Calmette-Guerin (BCG) and diphtheria, tetanus, and pertussis, and inactivated polio vaccine (DTP-IPV). BCG and DTP-IPV vaccines rarely cause psoriasis. The first case of psoriatic skin lesions after BCG vaccination was reported in the 1950s, and a few similar cases have been reported [9]. Here, we report a case of psoriatic skin lesions following BCG vaccination in a five-month-old girl with LTBI.

## Case presentation

A five-month-old female child received the BCG vaccine and a third dose of DTP-IPV at five months of age in accordance with the vaccination schedule recommended in Japan. Soon after the vaccination, erythema with pustulation developed at the BCG injection site. Erythematous plaques were also seen mainly on the trunk. Her father had suffered from tuberculosis when the patient was born, and her mother had LTBI. Therefore, the skin rash on the vaccination site was considered Koch’s phenomenon. She was presented at the pediatric department to check the infection status of tuberculosis. Blood tests at this initial visit showed increased white blood cell and platelet counts (Table 1).

**Table 1:** Blood test results

Tests	Reference Range	Patient Values
Hemogram		
White blood cells (/μL)	3500～9200	19340
Red blood cells (x10^6^/μL)	3.84～5.54	4.94
Platelets (/μL)	155,000-365,000	642,000
Hemoglobin (g/dL)	11.3～16.6	11.7
Electrolyte		
Sodium (mEq/L)	136-146	139
Potassium (mEq/L)	3.5-5.4	4.6
Chloride (mEq/L)	96-108	107
Renal function		
Blood urea nitrogen (mg/dL)	8～22	4.2
Creatinine (mg/dL)	0.35-1.11	0.20
Liver function		
Total bilirubin (mg/dL)	0.3-1.2	0.4
Aspartate transaminase (U/L)	8-38	37
Alanine transaminase (U/L)	4-44	26
γ-glutamyl transpeptidase (U/L)	16～84	14
Nutrition and inflammation and others		
Total protein (g/dL)	6.3～8.1	6.5
Albumin (g/dL)	3.9～5.1	3.9
Lactate Dehydrogenase (U/L)	119-229	300
Creatine kinase (U/L)	61-255	70
C-reactive protein (mg/dL)	～0.3	0.28

**Figure 1:**
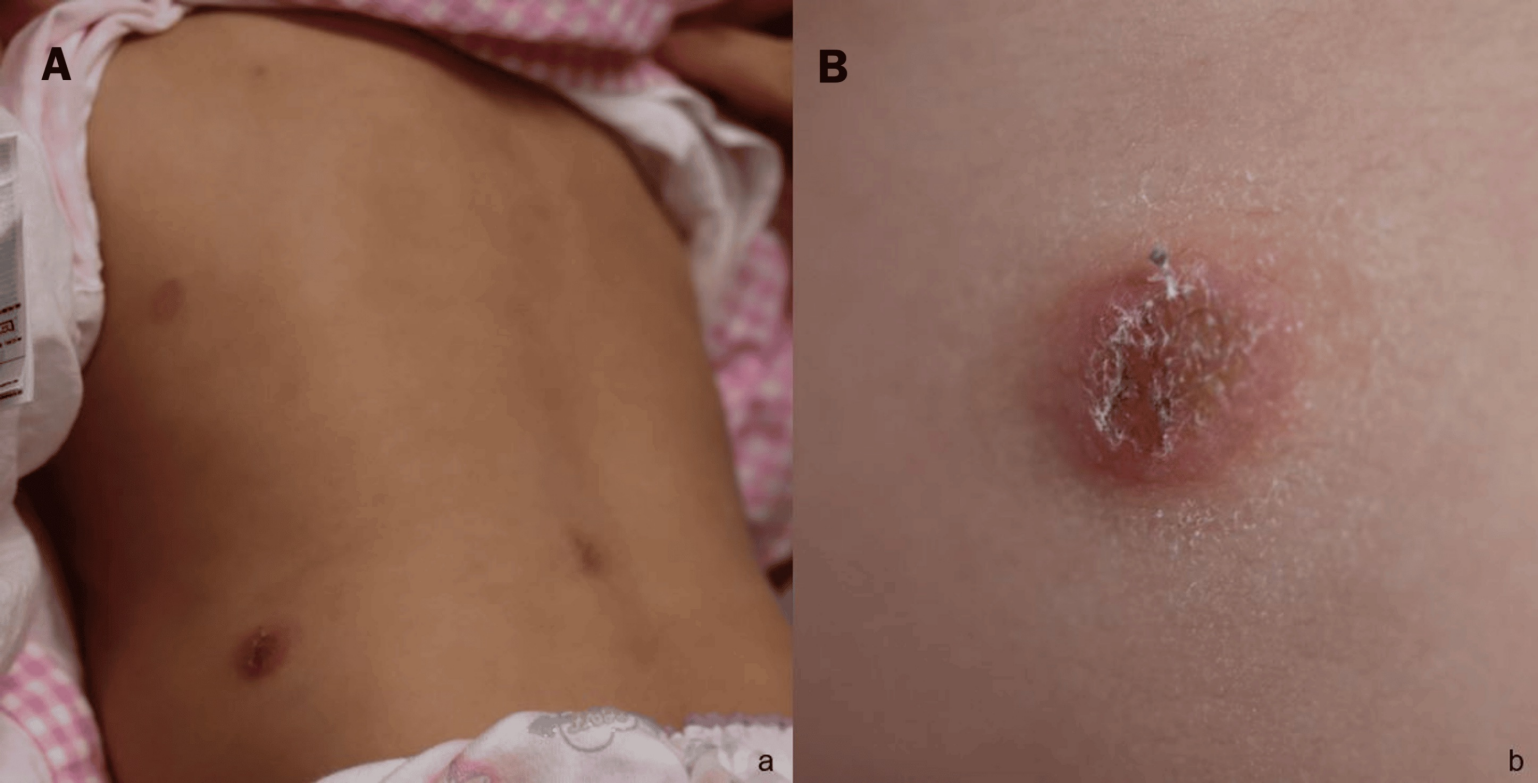
Clinical images showing (A) erythematous plaques scattered on the trunk and (B) a scaly erythematous plaque on the back

The tuberculin skin test was positive. A T-cell-based enzyme-linked immunospot assay for tuberculosis was also positive, while there were no abnormalities in the lung field, leading to the diagnosis of LTBI. Treatment with isoniazid was started two weeks after the patient's initial visit. Erythematous plaques on the trunk persisted (Figure 1), and the patient was referred to our department six weeks after the first visit to our hospital to rule out cutaneous tuberculosis.

**Figure 2:**
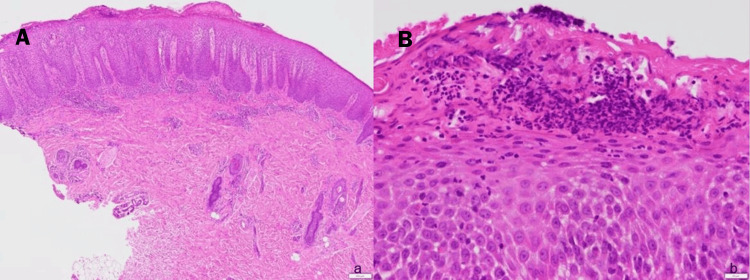
Histological images showing (A) Psoriasiform acanthosis and inflammatory infiltrate (H&E; x40) and (B) Munro’s microabscess (H&E; x400)

A skin biopsy revealed psoriasiform acanthosis and Munro's microabscess (Figure 2).

**Figure 3:**
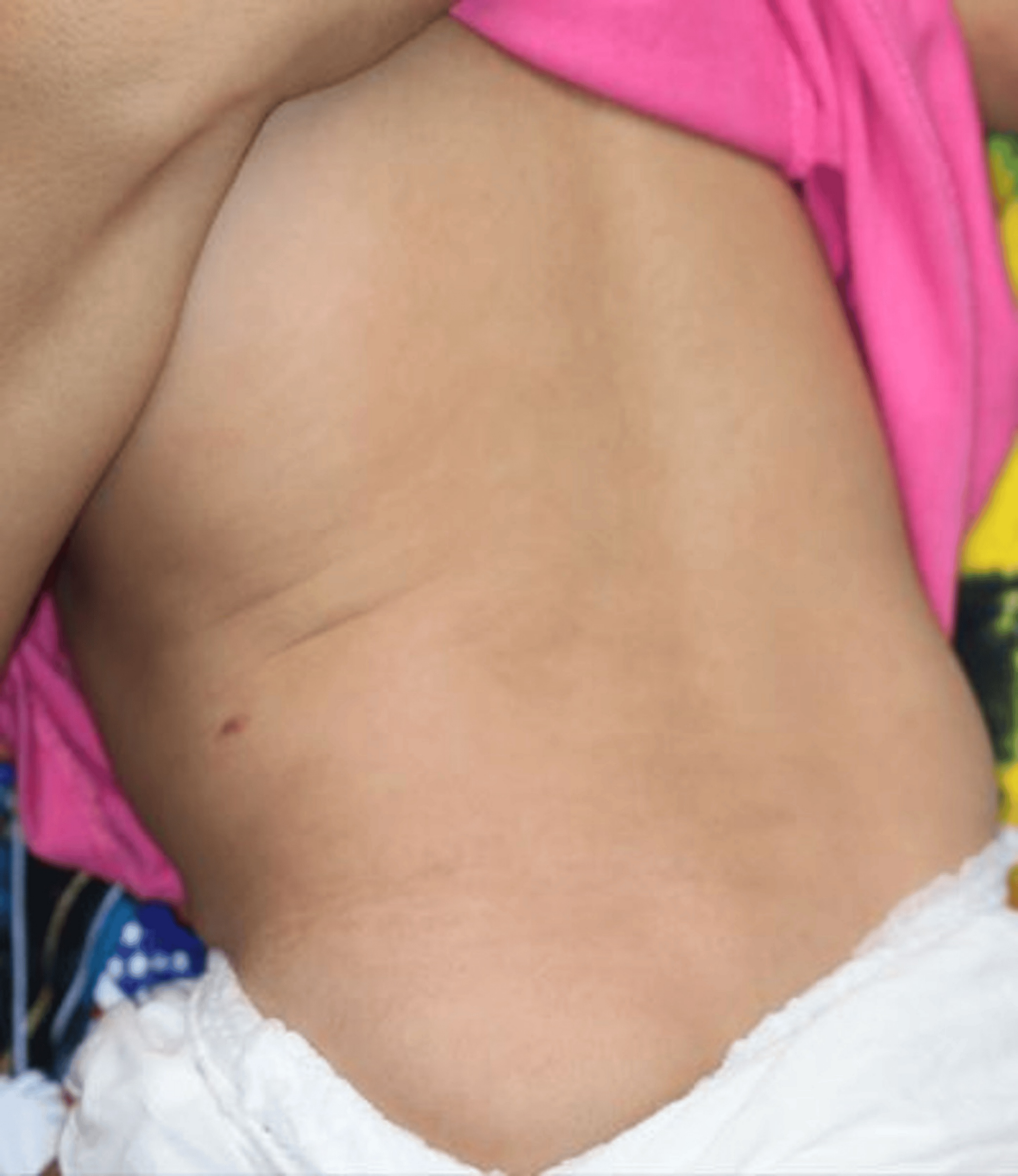
Clinical image three months after the start of treatment showing general resolution of psoriatic skin lesions

No mycobacteria were detected by Ziehl-Neelsen staining, and tissue culture was negative. Therefore, the patient was diagnosed with psoriatic skin lesions after BCG vaccination. Topical application of maxacalcitol 25 μg/g ointment and prednisolone valerate acetate 0.3% ointment relieved the plaques (Figure 3). No recurrence of skin rash was observed during a five-month follow-up.

## Discussion

The incidence of tuberculosis in Japan has been declining for the past several years. On the other hand, there are still more than 10,000 new cases or relapsed cases per year in the country [10]. There was only one reported case with psoriasis triggered by tetanus-diphtheria vaccination [11], while there were a few cases that developed psoriatic skin lesions after BCG vaccination [9,12,13]. To the best of our knowledge, only two cases of psoriasis-like skin rash in children after BCG vaccination have been reported [9,13] (Table 2).

**Table 2:** Summary of published cases of psoriatic skin lesion in children after Bacillus Calmette-Guerin (BCG) vaccination

Cases	Age	Sex	Type of psoriasis	Medical history	Treatment	Tuberculosis infection
Takayama et al. [9]	6 months	Fmale	Plaque, nail	Congenital mitral valve insufficiency	No specific treatment	None
Koca et al. [13]	7 years	Male	Guttate	None	Topical corticosteroid	None
Current case	5 months	Female	Plaque	Family history of TB (father) and a history of LTBI (mother)	Topical maxacalcitol	Latent tuberculosis infection

Each case has a different treatment strategy; In one, there was no specific treatment while in the other two, topical steroid or topical maxacalcitol were used [9,13]. There have been a few reports of psoriatic arthritis after BCG vaccination in adults [12], whereas there have been no reports of psoriatic arthritis in children. Although the mechanism by which the BCG vaccine causes psoriatic skin lesions remains unclear, mice vaccinated with BCG were reported to produce Th17 cytokines, which are important for the development of psoriasis [14]. Expression of various inflammatory cytokines, including tumor necrosis factor, is enhanced when the BCG vaccine is administered to patients previously infected with tuberculosis [15]. To the best of our knowledge, this is the first case with psoriatic skin lesions after BCG vaccination that was diagnosed with LTBI, which may have contributed to the development of skin eruptions. On the other hand, the high percentage of psoriasis patients with LTBI suggests that there may be many cases that have been missed [6].

## Conclusions

This report presented a case of psoriatic skin lesions caused by BCG vaccination in a pediatric patient with LTBI. To the best of our knowledge, this is the first case with psoriatic skin lesions after BCG vaccination that was diagnosed with LTBI. Since the incidence of psoriatic skin lesions after BCG vaccination is rarely reported despite the fact that BCG vaccination is a routine immunization in Japan, it is suspected that some trigger may be necessary for the development of psoriatic skin lesions after BCG vaccination. Although it is still unclear how psoriasis-like skin lesions develop after BCG vaccination, we hypothesize that LTBI may be a risk factor. We hope that this report will encourage doctors to consider the possibility of LTBI when they see a psoriasis-like skin rash in children.
